# Lead Isotope Characterization of Petroleum Fuels in Taipei, Taiwan

**DOI:** 10.3390/ijerph120504602

**Published:** 2015-04-24

**Authors:** Pei-Hsuan Yao, Guey-Shin Shyu, Ying-Fang Chang, Yu-Chen Chou, Chuan-Chou Shen, Chi-Su Chou, Tsun-Kuo Chang

**Affiliations:** 1 Agro-environment Laboratory (AELab), Department of Bioenvironmental Systems Engineering, National Taiwan University, Taipei City 106, Taiwan; E-Mail: d97622006@ntu.edu.tw (P.-H.Y.); 2 Department of Travel and Ecotourism, Tungnan University, New Taipei City 222, Taiwan; E-Mail: gsshyu@mail.tnu.edu.tw; 3 Green Energy and Environment Research Laboratories, Industrial Technology Research Institute, Hsinchu County 310, Taiwan; E-Mail: changyingfang@itri.org.tw; 4 High-Precision Mass Spectrometry and Environmental Change Laboratory (HISPEC), Department of Geosciences, National Taiwan University, Taipei City 106, Taiwan; E-Mails: r99224105@ntu.edu.tw (Y.-C.C.); river@ntu.edu.tw (C.-C.S.); 5 Ecological Engineering Research Center, National Taiwan University, Taipei City 106, Taiwan; E-Mail: chisuchou2012@gmail.com

**Keywords:** stable lead isotopes, environmental forensics, petroleum fuels.

## Abstract

Leaded gasoline in Taiwan was gradually phased out from 1983 to 2000. However, it is unclear whether unleaded gasoline still contributes to atmospheric lead (Pb) exposure in urban areas. In this study, Pb isotopic compositions of unleaded gasolines, with octane numbers of 92, 95, 98, and diesel from two local suppliers in Taipei were determined by multi-collector inductively coupled plasma mass spectrometry with a two-sigma uncertainty of ± 0.02 %. Lead isotopic ratios of vehicle exhaust (^208^Pb/^207^Pb: 2.427, ^206^Pb/^207^Pb: 1.148, as estimated from petroleum fuels) overlap with the reported aerosol data. This agreement indicates that local unleaded petroleum fuels, containing 10–45 ng·Pb·g^−1^, are merely one contributor among various sources to urban aerosol Pb. Additionally, the distinction between the products of the two companies is statistically significant in their individual ^208^Pb/^206^Pb ratios (*p-value* < 0.001, t test). Lead isotopic characterization appears to be applicable as a “fingerprinting” tool for tracing the sources of Pb pollution.

## 1. Introduction

Lead (Pb) is a toxic but useful element for human and has four stable isotopes, ^204^Pb, ^206^Pb, ^207^Pb, and ^208^Pb. Besides the largest use of Pb for lead-acid batteries, it is also used in alloys, as an anti-knock additive in petrol, cables, chemicals, solders, *etc.* [1]. Leaded gasoline, with alkyllead compounds as antiknock additives, was a major source of human Pb exposure and environmental Pb pollution through the 1980s [1,2,3]. Developing countries have set up strict regulations to ban Pb additives, e.g., tetraethyl and tetramethyl Pb, in order to eliminate the release of Pb from gasoline combustion [1,3,4]. Lead isotopic signatures have been demonstrated to be reliable tracers for distinguishing different sources of local and global Pb pollution (e.g., Erel *et al.* [5], Keinonen [6], Monna *et al.* [7], Mukai *et al.* [8], Widory *et al.* [9], Zheng *et al.* [10]). Atmospheric Pb has various origins in addition to the natural background such as coal and gasoline combustion, industrial emissions, lead smelters, and waste incineration [7,9,11]. Isotopic techniques have been used on Pb related environmental studies. For example, Monna *et al.* [7] utilized Pb isotopes to conduct environmental forensic studies in France and UK. Their results revealed gasoline was the main source of Pb in airborne particulate matter in France and UK. Widory *et al.* [9] coupled Pb and Sr isotopes to characterize the origin of aerosols in Beijing, China, and Pb isotopic ratios suggested that metal refining plants were the major source of atmospheric Pb, followed by thermal power stations and other coal combustion process.

The phasing out of Pb in gasoline in Taiwan started in 1983. In 2000, the supply of leaded gasoline was officially discontinued. Unleaded gasoline, containing less than 0.0013 g/L of Pb, was introduced [12]. Chinese Petroleum Corporation, Taiwan (CPC) and Formosa Plastics Corporation (FPC) are the major supplier of petroleum fuels in Taiwan [13]. Taiwan relies heavily on imported crude oil which accounts for 99 % of nation’s crude oil needs. Taiwan’s petroleum products follow a simplified process of crude oil unloading, refining, blending, transportation, and filling at service stations. By the end of 2012, 2668 gas stations were in service [14]. Also, more than 22.3 million on-road vehicles were operated in Taiwan, a number that almost doubled in the last two decades [14]. Atmospheric Pb concentrations in Taipei declined from 700 ng/m^3^ in 1991 [15] to 34 ng/m^3^ in 2002–2003 [16] and 27 ng/m^3^ in 2003–2004 [17]. Cord blood Pb levels decreased from 74.8 ± 22.5 µg/L in 1985–1987 [18] to 12.6 ± 1.8 µg/L in 2004–2005 [19]. These monitoring results indicate that the human exposure to environmental Pb in Taiwan has declined since the use of unleaded gasoline.

Hsu *et al.* [16] suggested that high level of atmospheric Pb in Taipei city, Taiwan might have occurred during the winter, under the influence of the northeast monsoon. They also demonstrated the impact of dust from China through the analysis of air mass trajectory and seasonal variations of Pb isotopic ratios in Taipei aerosols [17]. However, only particulate samples from the main tunnels in Taipei have been compared, and there was no direct data from fuel products or vehicle exhaust analysis. In another study, Pb isotopic ratios of aerosols in Pengjia Islet, where fuel combustion was negligible, have shown seasonal variations [20], but the Pb origins were not clear.

Since no isotopic fractionation occurs during any refining process, Pb isotopic characteristics are specific and unchanged for a given crude oil, fuel additive, gasoline, or Pb ore [21]. Although Pb from fuel combustion has been considered as a contributor to urban atmosphere in previous studies, direct Pb isotopic ratios survey in petroleum fuel is still rare. Until now, only Pb isotopic determination in unleaded gasoline products of Israel [4] and USA [21] has already been reported, whereas Pb isotopes in petroleum products of Taiwan has never been reported. The purposes of this study were, first of all, to determine Pb isotopic signatures of local fuel products, and secondly, to compare these signatures with those of other potential sources of atmospheric Pb, through data obtained in an urban area and from an islet with negligible fuel combustion.

## 2. Materials and Methods

### 2.1. Sampling

In recent years, unleaded gasoline, #92, #95, #98, and diesel from Chinese Petroleum Corporation, Taiwan (CPC) and Formosa Plastics Corporation (FPC) are commercially available in Taiwan [13]. The two suppliers use different dyestuffs for products identification. The color of #92 unleaded gasoline is green, while #95, #98 and diesel from CPC are yellow, red and yellow, respectively. FPC’s products are blue (#92), light yellow (#95), red (#98), and yellow (diesel). Fuel products were collected from service stations in metropolitan Taipei in 2012 (modified from Monna *et al.* [7]). After cleaning the gas pump nozzle dispenser with Kimwipes, each fuel sample was directly filled into an acid-clean 250 mL Teflon bottle and stored before chemical analysis.

### 2.2. Chemistry

Water was purified using a tandem ultrapure water system with Millipore Milli-Q ACADEMIC and Milli-Q ELEMENT systems. Teflon labware was acid-cleaned. All reagents were of ultrapure grade. Chemical procedure and instrumental analyses were performed on benches with class-100 laminar-flow air in a class-10000 clean room (Shen *et al.* [22]).

Sample treatment was done according to the following modified Monna’s method [7]. Gasoline sample (30 g; 0.3 g for diesel) was kept in a 30 mL Teflon vial and slowly evaporated at elevated temperature up to 150 °C on a hotplate for 5 h. The residue was digested with 2 mL 14N HNO_3_ and 0.2 mL 30% H_2_O_2_ at 160 °C with reflux. Then, dried and re-dissolved in 2 mL 5% HNO_3_. Recovery was 99.8 %–103.7 %. The solution was separated into two aliquots (w/w = 1:3), one for detecting Pb content and the other for further Pb separation and isotopic determination. Lead separation process was conducted with Eichrom 100–150-mesh Sr-Spec resin using the method modified from Gale [23]. Overall procedural Pb blank was 20 pg. Lead mass loss was < 10 %. 

Lead concentration was determined by a calibration plot, established with Merck Multi-Element Standards, on an inductively-coupled plasma-sector field mass spectrometer (ICP-SF-MS), Element II, Thermo Fisher (Bremen, Germany), with a detection limit of 0.001 ppb. The relative percent differences (RPD) were < 7 %. Lead isotopic ratios were determined by a multi-collector ICP-MS (MC-ICP-MS; Neptune, Thermo Fisher), equipped with a dry introduction device (Cetac Aridus, Teledyne CETAC Technologies, NE, USA). Three isobaric interferences, ^204^Hg, WO and ReO, were reported [24]. The intensities of ion beams of WO^+^ and ReO^+^ can be eliminated through chromatographic separation. An isobaric interference of ^204^Hg was only 1000 counts per second (cps), considered as insignificant. Thallium (Tl) isotopes were adopted for mass fractionation correction [24,25,26,27]. Lead isotopic ratios were normalized to ^205^Tl/^203^Tl = 2.3889 exponentially [24,28]. Replicate measurements made with 2–10 ng Pb for an international standard NIST SRM 981 showed the 2-sigma reproducibility of ± 0.02 % (Table 1). 

**Table 1:** Comparisons of precision and accuracy (NIST SRM 981; mean ± 2 S.D.).

Reference	^208^Pb/^206^Pb	^207^Pb/^206^Pb	^206^Pb/^204^Pb	Method
Baker [24]	2.1678 ± 0.0001	0.9149 ± 0.0004	16.942 ± 0.001	DS, MC-ICP-MS
	2.1678 ± 0.0002	0.9149 ± 0.0004	16.94 ± 0.01	Tl, MC-ICP-MS
Galer and Abouchami [29]	2.1677 ± 0.0001	0.91475 ± 0.00004	16.941 ± 0.001	TS, TIMS
Thirlwall [30]	2.1677 ± 0.0002	0.91483 ± 0.00006	16.941 ± 0.001	DS, TIMS
Thirlwall [28]	2.1677 ± 0.0002	0.91488 ± 0.00008	16.942 ± 0.003	DS, MC-ICP-MS
Weiss *et al.* [26]	2.1677 ± 0.0006	0.91404	16.947 ± 0.008	Tl, MC-ICP-MS
White *et al.* [27]	2.1646 ± 0.0008	0.9148 ± 0.0001	16.941 ± 0.004	Tl, MC-ICP-MS
This study	2.1675 ± 0.0003	0.9148 ± 0.0001	16.941 ± 0.008	Tl, MC-ICP-MS

DS: ^204^Pb-^2207^Pb double spike, Tl: Tl correction, TS: ^204^Pb-^206^Pb-^207^Pb triple spike, MC-ICP-MS: multi-collector ICP-MS, TIMS: thermal ionization mass spectrometry.

### 2.3. Environmental Impact

Acids are toxic, corrosive reagents that can burn skin and damage respiratory organs. Therefore, waste acid was neutralized before discharge. Unused fuel products were recycled.

## 3. Results

### 3.1. Lead Level of Taiwan’s Fuel Products

**Figure 1:**
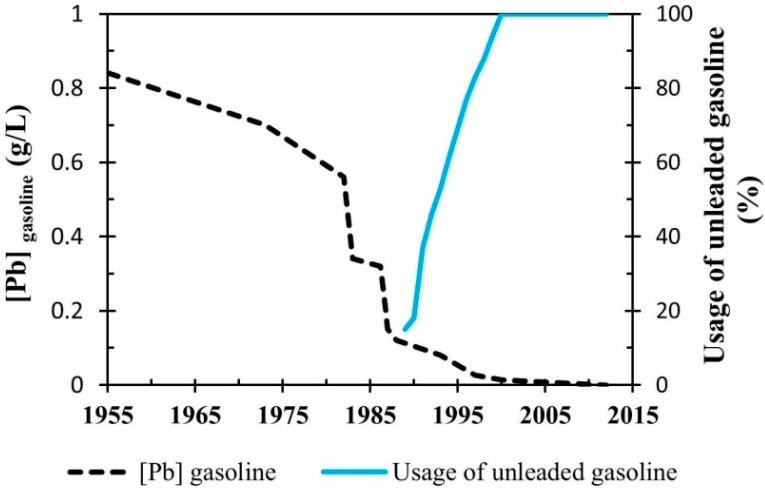
History of the use of petroleum fuels in Taiwan (data from [12]).

Results of Pb isotopic ratios and concentrations for Taiwan’s fuel products are summarized in Table 2. Lead concentrations in fuel products of Taiwan range from 9.6 ng·g^−1^ to 17.9 ng·g^−1^ (unleaded gasoline) and 29.4 ng·g^−1^ to 44.8 ng·g^−1^ (diesel), below the national guideline value of 0.013 g/L [12] (Table 2). After switching from leaded to unleaded gasoline (Figure 1), Pb in unleaded gasoline products (measured in ppb) is definitely not tetraethyl Pb. However, trace amount of Pb in these products might have originated from the crude oil. Diesel has elevated Pb level than unleaded gasoline. And CPC’s #92 unleaded gasoline has the lowest Pb content, 9.6 ng·g^−1^.

**Table 2:** Summary of Pb isotopic characteristics and level in Taiwan’s fuel products (mean ± 1 S.D., n = 3).

Brand	Product	^208^Pb/^207^Pb	^208^Pb/^206^Pb	^206^Pb/^207^Pb	^206^Pb/^204^Pb	^20^^7^Pb/^204^Pb	^20^^8^Pb/^204^Pb	[Pb] (ng·g^−1^)
Chinese Petroleum Corporation, Taiwan (CPC)	#92 unleaded gasoline	2.422 ± 0.002	2.111 ± 0.002	1.147 ± 0.002	17.86 ± 0.02	15.57 ± 0.01	37.71 ± 0.02	9.6 ± 3.2
	#95 unleaded gasoline	2.429 ± 0.003	2.110 ± 0.005	1.151 ± 0.002	17.94 ± 0.02	15.588 ± 0.006	37.86 ± 0.06	17.9 ± 16.9
	#98 unleaded gasoline	2.417 ± 0.003	2.107 ± 0.004	1.147 ± 0.003	17.87 ± 0.05	15.578 ± 0.003	37.65 ± 0.04	16.4 ± 2.6
	diesel	2.431 ± 0.004	2.115 ± 0.009	1.149 ± 0.007	17.89 ± 0.08	15.57 ± 0.02	37.84 ± 0.06	44.8 ± 75.2
Formosa Plastics Corporation (FPC)	#92 unleaded gasoline	2.4288 ± 0.0009	2.1204 ± 0.0004	1.1454 ± 0.0004	17.836 ± 0.004	15.571 ± 0.007	37.819 ± 0.004	17.1 ± 3.5
	#95 unleaded gasoline	2.425 ± 0.001	2.125 ± 0.004	1.141 ± 0.002	17.78 ± 0.05	15.58 ± 0.01	37.79 ± 0.04	14.6 ± 3.9
	#98 unleaded gasoline	2.4278 ± 0.0009	2.1192 ± 0.0008	1.1456 ± 0.0002	17.847 ± 0.008	15.579 ± 0.006	37.822 ± 0.002	15.9 ± 2.4
	diesel	2.433 ± 0.003	2.119 ± 0.005	1.148 ± 0.003	17.89 ± 0.05	15.582 ± 0.004	37.91 ± 0.04	29.4 ± 22.4

### 3.2. Lead Isotopic Ratios of Taiwan’s Fuel Products

**Figure 2:**
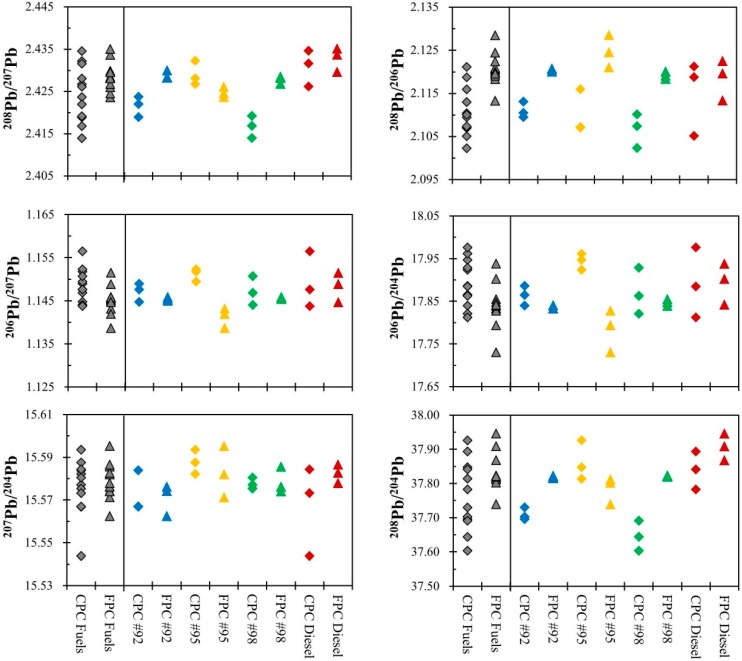
Lead isotopic ratios of Taiwan’s fuel products collected from Chinese Petroleum Corporation, Taiwan (CPC) and Formosa Plastics Corporation (FPC). Besides, #92, #95 and #98 represent unleaded gasoline with octane number 92, 95 and 98, respectively.

Our unleaded gasoline and diesel samples contain Pb in parts per billion (ppb) levels, which were analyzed by high-precision Pb isotopic analysis. The results of Pb isotopic ratios for Taiwan’s fuel products are given in Table 2 and in Figure 2. Monna *et al.* [7] pointed out that environmental scientists tend to use ^206^Pb/^207^Pb when conducting environmental forensics. With ^206^Pb/^207^Pb data, identification of gasoline as the dominant source of environmental Pb can be readily achieved [31]. Our measurements shows CPC’s #95 unleaded gasoline has the highest ^206^Pb/^207^Pb ratios, averaging 1.151 (n = 3), while FPC’s #95 unleaded gasoline has the lowest mean of 1.141 (n = 3). 

While Monna *et al.* [7] applied ^206^Pb/^207^Pb *versus* ^208^Pb/^206^Pb diagrams and ^206^Pb/^207^Pb *versus* ^206^Pb/^204^Pb diagram in conducting European environmental forensics, the available Pb isotopic records for Taiwan (*i.e.*, [17,20]) were just given in ^208^Pb/^207^Pb and ^206^Pb/^207^Pb ratios. Because ^204^Pb is not readily detected by ICP-Q-MS, many previous environmental studies do not present it, and we would lack interpretation on mixtures from more than two sources [31]. Therefore, we took ^208^Pb/^207^Pb and ^206^Pb/^207^Pb values for comparison with recent studies in the following discussion and presented all ^204^Pb ratios (Table 2) for further studies to refer. 

## 4. Discussion

### 4.1. Comparison on Pb Isotopic Ratios of Worldwide Petroleum Fuels

Petroleum fuel combustion has been considered as a major Pb input to the urban atmosphere, especially in the 1980s. But, rather than direct Pb isotopic ratios survey in fuels, most were obtained indirectly through calculating Pb-ores data or sampling of aerosols. Moreover, available data in Asia are limited. In Table 3, we have compared our Pb isotopic ratios with those measured directly in other countries, including Austria [32], Finland [6], France [7], Germany [33], Hungary [32], Israel [5], Mexico [34], The Netherlands [32], Poland [32], Russia [8], Switzerland [11], UK [7,33,35], and USA [21,36]. Unleaded gasoline products were only obtained from Israel and USA, and the rest are all leaded products.

As observed in Table 3, Pb isotopic ratios vary in region, period and type, *i.e.*, leaded or unleaded. Leaded gasoline products in Israel, France and UK have similar Pb isotopic compositions, which were characterized by the added alkyl-lead from a major producer in Europe [5,7]. The range of ^206^Pb/^207^Pb ratio is quite distinguishable among unleaded gasoline sold in Israel (1.11–1.15), Taiwan (1.14–1.15) and USA (1.19–1.24), although there is a little overlap in the former two. Besides, diesel in Taiwan has much higher (more radiogenic) ^206^Pb/^207^Pb ratios than diesel in Switzerland (Table 2). The variation spreads were due to the different crude oil types and fuel formulations. Since no Pb-contained additive is added, lead isotopic ratios of unleaded gasoline products reflect Pb isotopic composition of crude oil. 

**Table 3:** Summary of Pb isotopic characteristics in global petroleum products.

Country	Item	Year	^208^Pb/^206^Pb	^206^Pb/^207^Pb	^206^Pb/^204^Pb	Reference
Austria	leaded gasoline	1990	NA	1.111	NA	Hopper *et al.* [32]
Finland	gasoline	1987	NA	1.122–1.159	17.42–18.00	Keinonen [6]
France	gasoline	1995	2.172–2.198	1.069–1.094	16.56–17.07	Monna *et al.* [7]
Germany	leaded gasoline	NA	NA	1.10	NA	Krause *et al.* [33]
Hungary	leaded gasoline	1990	NA	1.072	NA	Hopper *et al.* [32]
Israel	91 gasoline	1995	2.148–2.158	1.094–1.110	NA	Erel *et al.* [5]
	96 gasoline	1995	2.142–2.147	1.108–1.119	NA	
	unleaded gasoline	1995	2.112–2.142	1.108–1.146	NA	
Mexico	leaded gasoline	1988–1989	2.049–2.055	1.202–1.204	18.69–18.73	Sañudo-Wilhelmy and Flegal [34]
Netherlands	leaded gasoline	1990	NA	1.062	NA	Hopper *et al.* [32]
Poland	leaded gasoline	1988	NA	1.174	NA	Hopper *et al.* [32]
Russia	leaded gasoline	NA	2.117–2.129	1.135–1.149	NA	Mukai *et al.* [8]
Switzerland	leaded petrol	1996–1997	2.116–2.179	1.075–1.358	16.59–17.63	Chiaradia and Cupelin [11]
	diesel	1997	2.146	1.110	17.29	
UK	leaded gasoline	1989–1998	NA	1.056–1.098	NA	Farmer *et al.* [35]
		NA	NA	1.07	NA	Krause *et al.* [33]
		1994	1.189–2.197	1.059–1.079	16.50–16.72	Monna *et al.* [7]
USA	leaded gasoline	1964	NA	1.115–1.160	17.38–18.14	Chow and Johnstone [36]
	unleaded gasoline	1997–1999	NA	1.190–1.240	18.40–19.50	Hurst [21]
Taiwan	unleaded gasoline	2012	2.102–2.129	1.139–1.152	17.73–17.96	This study
	diesel	2012	2.105–2.122	1.144–1.157	17.81–17.98	

NA: not mentioned.

### 4.2. Estimation on Taiwanese Local Vehicle Emissions

As depicted in Figure 2, fuel products available from the two major suppliers have significant different ^208^Pb/^206^Pb (*p-value* < 0.001, t test). #95 unleaded gasoline was also found as the obvious distinctive product. The significant difference between the ^208^Pb/^206^Pb of petroleum fuel products from CPC and FPC has revealed a new indicator for further petroleum-related environmental forensics. 

Based on the number of service stations in Taipei, the market share of CPC and FPC were 75 % (n = 57) and 25 % (n = 19), respectively [13]. Meanwhile, the sales volume of unleaded gasoline #92, #95, #98, and diesel were 25 %, 35 %, 10 %, and 30 %, respectively. Based on the sales and market share, Taiwanese local vehicle emissions could be estimated as 2.427 for ^208^Pb/^207^Pb, 2.114 for ^208^Pb/^206^Pb, 1.148 for ^206^Pb/^207^Pb, 17.89 for ^206^Pb/^204^Pb, 15.58 for ^20^^7^Pb/^204^Pb, and 37.81 for ^20^^8^Pb/^204^Pb.

### 4.3. Environmental forensic Application in Taipei City’s Atmosphere

Taipei city has an area of 271.8 km^2^ with a population of 2.6 million. In Taipei, the Rapid Transit System transports 1.6 million passengers per day in 2012, but there are 1.8 million motor vehicles in the city, and 1.6 million passengers use city buses per day. During the period before phasing out Pb from gasoline, atmospheric Pb concentrations in Taipei once reached 700 ± 390 ng/m^3^ [15]. Airborne Pb levels recently decreased to less than 27 ± 61 ng/m^3^ [17]. During a period from April 2003 to February 2004, ^208^Pb/^207^Pb ratios of Taipei’s aerosols ranged from 2.35 to 2.45, and ^206^Pb/^207^Pb ratios from 1.12 to 1.17 [17].

**Figure 3:**
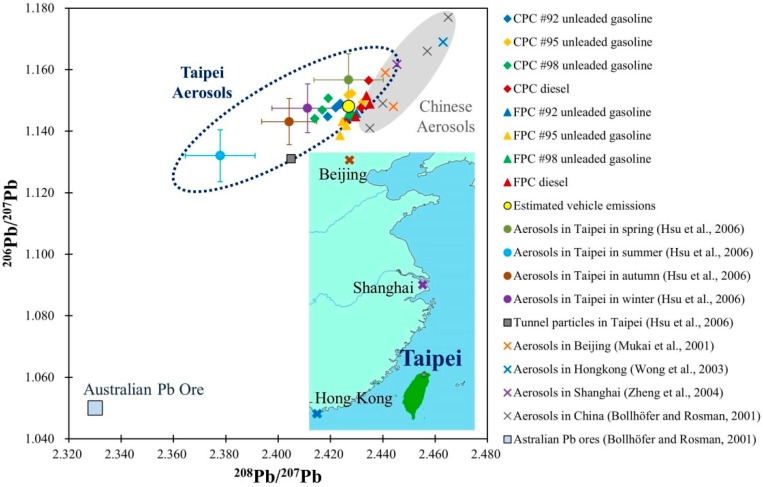
Scatter plot of ^208^Pb/^207^Pb *versus* ^206^Pb/^207^Pb for fuel products, estimated local vehicle emissions (the yellow circle), and particulate matter accumulated on wall tile surface from the main tunnels (PMT samples) [17] (the dark gray square) and seasonal aerosols collected in Taipei (drawn as the dotted ellipse; refer to Hsu *et al.* [17] for details data). The gray ellipse area represents Chinese aerosols [10,38,44,45]. Lead isotopic feature of Australian Pb ore [44], as the major import into Taiwan for industry, is also shown.

Lead levels in the air and blood have significantly decreased in the cities, due to the phase-out of leaded gasoline [3,18,19,37]. Taipei is in a basin terrain with heavy traffic, so the traffic-related contribution cannot be excluded. According to our previous estimation, the ^208^Pb/^207^Pb and ^206^Pb/^207^Pb values of Taiwan’s local vehicle exhaust were 2.427 and 1.148, respectively, which are close to that of Taipei’s aerosols reported by Hsu *et al.* [17], but the isotopic fingerprints of Taipei’s aerosols [17] and our estimated local vehicle exhaust or fuel products are not in a perfect match (Figure 3). The former with an extensive and seasonal variation in its isotopic characteristics implies that multiple Pb sources exist in Taipei.

As illustrated in Figure 3, our estimated Taiwanese local vehicle emissions and Taipei’s aerosols in winter and spring times [17] have similar Pb isotopic ratios, while Taipei’s aerosols in the spring also show a little overlap with aerosols in Beijing and Shanghai, China [10,38]. However, the above measurements are not performed in the same period. Aerosols exhibit Pb isotopic ratios from fuel combustion since the atmospheric dispersion condition is poor in the winter. Another Pb contributor that could affect Taiwan is the long-range transport of dust from China during the northeastern monsoon seasons from December to next May, especially in February, March and April [39]. 

Aerosols in the summer showed different isotopic patterns compared to our measurements on fuel products, which suggests that other main contributors might also exist. Resuspension of Pb-tainted urban soil, *i.e.*, the contaminant remained from leaded gasoline consumption, could pose a threat on the current urban environment [37,40,41]. In an earlier study, Hsu *et al.* [17] collected and analyzed particulate matter accumulated on wall tile surface (PMT) from the main tunnels in downtown Taipei and assumed the Pb originated from the local vehicle emissions. However, their reported values, 2.405 for ^208^Pb/^207^Pb and 1.131 for ^206^Pb/^207^Pb, were lower than ours. We suggest that the PMT samples should be explained as the combustion products of leaded gasoline coated on tunnel wall, which influence Taipei’s aerosols in the summer and autumn seasons, consistent with Laidlaw’s [37] finding. Lead from the past leaded gasoline continues to affect the current urban air through resuspension during dry seasons with high evapotranspiration rates [37]. Even though, urban traffic exhausts alone is insufficient to form the summer aerosol Pb isotopic characteristic.

Flegal *et al.* [42] have summarized the major sources of Pb contamination in China, including: (1) deposits from previous emissions of leaded gasoline, (2) previous and continuing emissions from fossil fuel combustion, (3) previous and continuing emissions from other industrial activities, and (4) previous and continuing additions of contaminated fertilizers, sewage, and untreated wastewater to agricultural fields. However, unlike China, coal combustion almost exclusively takes place in five thermal power plants in Taiwan [14] and the coals are mainly imported from Australia and Indonesia, which have a higher ^206^Pb/^207^Pb ratio of 1.21 and 1.18, respectively [43]. Meanwhile, Taiwan also imports Australian Pb ores, which have low ^208^Pb/^207^Pb and ^206^Pb/^207^Pb values of 2.33 and 1.05 [44]. Therefore, the lowest ^206^Pb/^207^Pb signature found in the summer is believed to have come from pervious and current urban traffic exhausts mixed with southwest outflow of Taiwanese industrial processes and coal-powered electricity generators. To sum up, local vehicle fuel combustion and industrial activities, as well as the long-range dust transport from China in the specific seasons have dominated isotopic characteristics of aerosols in Taipei metropolis (Figure 3).

### 4.4. Environmental Forensic Application in Pengjia Islet’s Atmosphere

Another available Pb isotopic record in Taiwan concerns Pengjia Islet’s airborne particulate material, surveyed by Lien [20]. Pengjia Islet (25°37ʹ55ʺ N, 122°04ʹ20ʺ E) is located 56 km off Keelung Harbor, in northern Taiwan. With an area of 114 hectares, the islet has a highest altitude of 165 m, gradually decreasing from east to west. Due to the fierce wind its vegetation consists primarily of grasses. Only a few people, less than 40, are stationed on the islet, such as the staff of the lighthouse, weather station and some soldiers. The islet is administered by the Keelung Municipal Government. In addition, its aerosols show seasonal differences in Pb isotopic ratios during 1997 to 2000 [20].

**Figure 4:**
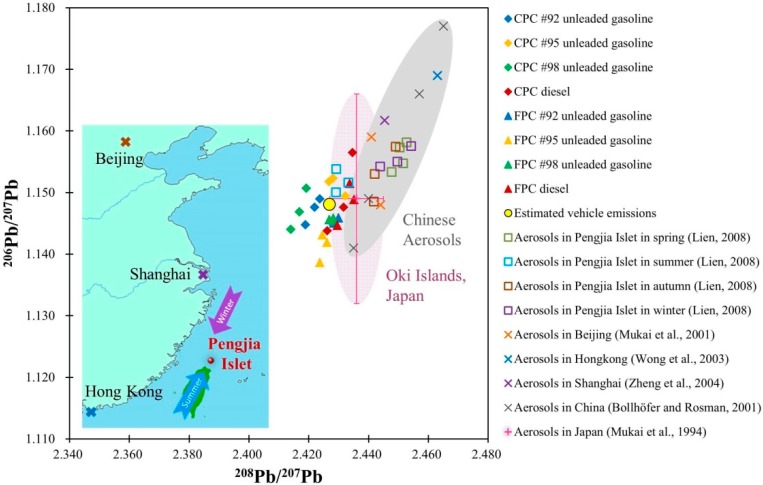
Scatter plot of ^208^Pb/^207^Pb *versus* ^206^Pb/^207^Pb for fuel products, estimated Taiwanese vehicle emissions (the yellow circle), together with the seasonal aerosols collected in Pengjia Islet by Lien [20], drawn as blank squares. The gray ellipse area represents Chinese aerosols [10,38,44,45]. In addition, aerosol records in Oki Islands, Japan [8] recalculated and summarized are also shown as a pink ellipse.

Mukai *et al.* [8] suggested that the Pb isotopic signatures of aerosols are a good indicator of long-range transport of air pollutants in Asia, with a demonstration in Oki Islands, Japan. Figure 4 illustrates ^208^Pb/^207^Pb *versus* ^206^Pb/^207^Pb plot of aerosols in Pengjia Islet [20] and its possible Pb sources. In addition to our measurements, we also examined aerosol records in Beijing, Hong Kong and Shanghai, China [10,38,44,45], and Oki Islands, Japan [8]. Because of its clean environment and small population, we assumed that the local Pb contribution is negligible. As displayed in Figure 4, Pb isotopic ratios of aerosols in the summer show distinctive characteristics from those of other seasons in Pengjia Islet but similar to the isotopic ratios of our Taiwanese local vehicle emissions. Pb isotopic compositions of aerosols in Pengjia Islet overlap and resemble the pattern of Chinese aerosols [10,38] in seasons other than summer, which might be caused by different seasonal wind directions. Fuels’ Pb isotopic ratios and historical Pb isotopic data in Pengjia Islet suggest that the Pb in aerosols was more likely from fuel combustion in Taiwan in the summer months, when the southwest monsoon prevails, while in the northeast monsoon season, the long-range transport of Pb-rich aerosols from China becomes dominant (Figure 4). 

### 4.5. Implication and Limitation

Because Taiwan imports most of its crude oil, Pb isotopic ratios in Taiwan’s petroleum products might change with the supply sources. However, gasoline and diesel products from the two local major suppliers have distinctive ^208^Pb/^206^Pb values, which can help identify the sources of petroleum fuels accidentally released into the environment, and trace the liability in oil spills. Tracing anthropogenic air pollution can be achieved by comparing our measurements with previous atmospheric studies in Taiwan and neighboring countries. For example, the deciphering Pb isotopic ratios of atmospheric particles in Pengjia Islet may be useful to study the impact of long-range dust transport from China on northern Taiwan. 

Lead isotopic ratios show greater seasonal variations in Taipei city (ICP-Q-MS data [17]) than in Pengjia Islet (MC-ICP-MS data without ^204^Pb ratios [20]). This implies that in addition to local fuel combustion (according to our own MC-ICP-MS data), multiple Pb contributors exist in metropolitan Taipei. A binary mixing model [7,17] could not be applied in this study to calculate the relative contribution due to the complex constituents and poor linearity. Coal and Pb ores for electricity and industry are mainly imported from Australia. According to the isotopic characteristics, air quality in Taipei metropolis is affected by local fuel combustion, resuspended solid particles (previously assumed as PMT samples), southwest airflow from electric power plants, as well as dust from China through the northeastern monsoon. Among those contributors, resuspended solid particles and air pollutants from China mainland pose pollution threats and merit further investigation. 

Laidlaw *et al.* [37], Harris and Davidson [40] and Zahran *et al.* [41] have highlighted the role of resuspended Pb-tainted urban soil, which had been contaminated by the past leaded gasoline emissions, as a dominant source of atmospheric Pb in cities. During dry seasons when evapotranspiration potential is high and soil moisture is low, Pb from the past contaminated soil continues to affect the current urban air [37]. The more Pb-tainted soil is resuspended, the higher the Pb level that is monitored in the air [37,41]. Furthermore, the legacy of Pb in urban atmospheres cannot hide under the novel isotopic finger-printing technique. After the phase-out of leaded gasoline, other contributors to air pollution have been uncovered by distinctive Pb isotopic signatures, and even the isotopic ratios of unleaded fuel products can be used for tracking. Environmental Pb and its isotopic characteristics will be shown in human teeth and bones as a record on Pb exposure as a georeferencing means as suggested by Kamenov and Gulson [46]. Besides petroleum fuel combustion in metropolis, Pb isotopic fingerprinting could identify and perhaps quantify contributions from complex domestic sources and cross-border transport of air pollutants. Through improved analytical protocols and instruments [23,24,25,26,27,28,29,30], reliable ^204^Pb (non-radiogenic but not readily analyzed in the past) data obtained could help to characterize and differentiate mixed multiple sources [31]. Further investigations on high-precision Pb isotopic ratios of Pb pollution sources and other related media are merited.

## 5. Conclusions

The following conclusions are based on this study and the analysis of related investigations:

(1) Urban aerosol Pb content has decreased by almost one order of magnitude compared to levels in 1991 when leaded fuels were still in use.
(2) Precise Pb isotopic ratios of unleaded petroleum fuels in Taipei, Taiwan were determined and thoroughly analyzed, which is the first such attempt in East Asia. Our results show that lead isotopic characterization is applicable as a “fingerprinting” tool for tracing Pb pollution sources. The distinction between the products of the two oil companies is statistically significant (*p-value* < 0.001, t test) in their individual ^208^Pb/^206^Pb ratios. Lead isotopic ratios of vehicle exhaust (^208^Pb/^207^Pb: 2.427, ^206^Pb/^207^Pb: 1.148, as estimated from petroleum fuels) overlap with the reported urban aerosol data.
(3) Fuel products and Taipei’s aerosols show similar isotopic signatures in the winter when poor atmospheric dispersion conditions persist. This suggests that local unleaded fuel combustion was a Pb contributor to the metropolitan air. Greater seasonal variation of Pb isotopic ratios in spring and summer time implies that other sources exist in addition to fuel combustion. Considering the climate conditions, Asian dust storms (in spring), resuspended solid or soil particles, and the southwest airflow of anthropogenic activities (in summertime) might be the key Pb contributors in air during the recent past.
